# Cross-sectional associations of objectively measured physical activity and sedentary time with sarcopenia and sarcopenic obesity in older men

**DOI:** 10.1016/j.ypmed.2016.08.040

**Published:** 2016-10

**Authors:** Daniel A. Aggio, Claudio Sartini, Olia Papacosta, Lucy T. Lennon, Sarah Ash, Peter H. Whincup, S. Goya Wannamethee, Barbara J. Jefferis

**Affiliations:** a UCL Department of Primary Care & Population Health, UCL Medical School, Rowland Hill Street, London NW3 2PF, UK; b UCL Physical Activity Research Group, UK; c Population Health Research Institute, St George's University of London, Cranmer Terrace, London. SW17 0RE, UK

**Keywords:** Physical activity, Sarcopenia, Sarcopenic obesity, Muscle mass

## Abstract

This study investigated associations between objectively measured physical activity (PA) with sarcopenia and sarcopenic obesity in older British men. Participants were men aged 70–92 years (n = 1286) recruited from UK Primary Care Centres. Outcomes included (i) sarcopenia, defined as low muscle mass (lowest two fifths of the mid-upper arm muscle circumference distribution) accompanied by low muscular strength (hand grip strength < 30 kg) or low physical performance (gait speed ≤ 0.8 m/s); (ii) severe sarcopenia, required all three conditions; (iii) sarcopenic obesity defined as sarcopenia or severe sarcopenia and a waist circumference of > 102 cm. Independent variables included time spent in PA intensities measured by GT3x accelerometers, worn during one week in 2010–12. Multinomial regression models were used for cross-sectional analyses relating PA and sarcopenia. In total, 14.2% (n = 183) of men had sarcopenia and a further 5.4% (n = 70) had severe sarcopenia. 25.3% of sarcopenic or severely sarcopenic men were obese. Each extra 30 min per day of moderate-to-vigorous PA (MVPA) was associated with a reduced risk of severe sarcopenia (relative risk [RR] 0.53, 95% confidence interval [CI] 0.30, 0.93) and sarcopenic obesity (RR 0.47 [95% CI 0.27, 0.84]). Light PA (LPA) and sedentary breaks were marginally associated with a reduced risk of sarcopenic obesity. Sedentary time was marginally associated with an increased risk of sarcopenic obesity independent of MVPA (RR 1.18 [95% CI 0.99, 1.40]). MVPA may reduce the risk of severe sarcopenia and sarcopenic obesity among older men. Reducing sedentary time and increasing LPA and sedentary breaks may also protect against sarcopenic obesity.

## Highlights

• Light and moderate-to-vigorous physical activity (MVPA) were measured objectively.
• Sedentary time (ST) and breaks in ST were measured objectively.
• MVPA was independently associated with risk of severe sarcopenia.
• MVPA and light PA were independently associated with risk of sarcopenic obesity.
• ST and breaks in ST were independently associated with risk of sarcopenic obesity.

## 1 Introduction

Normal aging involves important changes to body composition, including decreased muscle mass and increased fat mass (Zamboni et al., 2008). The age-related loss of muscle mass combined with loss of muscular strength and/or function is referred to as sarcopenia and occurs in up to 29% of community-dwelling older adults (Cruz-Jentoft et al., 2014). Sarcopenia is associated with an increased risk of frailty, disability and mortality in older adults (Waters et al., 2010, Landi et al., 2013). The co-occurrence of sarcopenia and obesity, defined as sarcopenic obesity, may heighten effects on health outcomes (Atkins et al., 2014a, Dominguez and Barbagallo, 2007, Zamboni et al., 2008). As physical activity (PA) is associated with lower fat mass and increased muscular strength and function (Bann et al., 2015, Shephard et al., 2013, Kuh et al., 2005), it may significantly reduce the risk of sarcopenia and sarcopenic obesity, making it one of the most important modifiable risk factors. Resistance training is particularly beneficial for improving muscular strength and function in the elderly (Cruz-Jentoft et al., 2014).

A number of cross-sectional and longitudinal studies have investigated the association between PA and sarcopenia using predominantly self-reported measures of PA. These studies have consistently shown that higher overall levels of PA are associated with a reduced risk of sarcopenia and sarcopenic obesity (Ryu et al., 2013, Kim et al., 2013, Atkins et al., 2014a, Hughes et al., 2001). One study reported that the risk of sarcopenia and sarcopenic obesity can be reduced by up to 74% and 73% in regularly active men, respectively (Ryu et al., 2013). Although self-report measures provide useful information on context of PA, objective measures allow more reliable estimates of volume and intensity. While studies using objective measures are few (Shephard et al., 2013, Bann et al., 2014, Park et al., 2010) they appear to confirm previous findings using self-reported PA. A recent prospective study showed that high levels of objectively measured walking approximately halved the risk of sarcopenia (Shephard et al., 2013). The authors also observed additional reductions in risk with PA at a moderate-to-vigorous intensity. Other studies report positive associations between PA and the individual components of sarcopenia, including muscle mass (Abe et al., 2012, Bann et al., 2014), muscular strength and function (Kuh et al., 2005, Eibich et al., 2015). To date, the association between specific intensities of objectively measured PA with both sarcopenia and sarcopenic obesity has not been well explored. Studies have not addressed whether differences in sedentary time, breaks in sedentary time and light PA (LPA) are associated with these conditions, which is important as older adults spend the majority of their time sedentary or in LPA. In addition, the association of PA with severe sarcopenia (low muscle mass accompanied by low muscular strength and physical performance (Cruz-Jentoft et al., 2010)) has not yet been investigated. Therefore the primary aim of this study was to investigate how objectively measured PA levels of different intensities are associated with sarcopenia, severe sarcopenia and sarcopenic obesity in community-dwelling older British men. We also investigated whether self-reported resistance training was associated with these conditions. Finally, we explored associations between different intensities of PA with the individual components of sarcopenia and sarcopenic obesity, namely muscle mass, physical performance, strength and obesity.

## 2 Methods

### 2.1 Participants

The British Regional Heart Study (BRHS) is an ongoing prospective cohort study involving 7735 men from 24 towns in Great Britain. The cohort were recruited from primary care practices and initially examined in 1978–80 aged 40 to 59 years. In 2010–12, all 3137 surviving cohort members aged 72–91 years were invited to attend a physical examination, complete a lifestyle and medical history questionnaire and have their PA levels measured objectively.

## 3 Exposure measures: physical activity

### 3.1 Accelerometry data

Participants were instructed to wear a GT3X accelerometer (Actigraph, Pensacola, Florida, USA) over the hip for 7 days, during waking hours, removing only for water-based activities. Accelerometer data were treated using standard techniques described previously (Jefferis et al., 2014). Non-wear time was identified and excluded using the R package “Physical Activity” (Choi et al., 2011). Ninety minute bouts or more of consecutive zero counts were classified as non-wear time; during these periods, intervals of up to 2 min of non-zero counts were also classified as non-wear time if no activity counts were detected during the 30 min before and after that interval, allowing for accidental movement when the accelerometer was not being worn. Raw data from the vertical axis were integrated into 60 s epochs and used to derive counts per minute (CPM). Time (minutes per day) spent sedentary and in different intensities of PA were derived using standard CPM-based intensity threshold values for older adults of < 100 for sedentary behaviour, 100–1040 for LPA and > 1040 for moderate-to-vigorous PA (MVPA). Valid data required ≥ 3 days of ≥ 600 min of wear time.

### 3.2 Self-reported PA data

Participants self-reported their habitual PA levels, including how often they make journeys by foot or by bike, recreational activities and participation in sports. A total PA score was derived based on the frequency and type of activity, which has previously been validated (Wannamethee et al., 2002). Men were grouped as inactive, occasional (regular walking or recreational activity only), light (more-frequent recreational activities, sporting exercise less than once a week, or regular walking plus some recreational activity), moderate (cycling, very frequent weekend recreational activities plus regular walking, or sporting activity once a week), moderately vigorous (sporting activity at least once a week or frequent cycling, plus frequent recreational activities or walking, or frequent sporting activities only) or vigorous (very frequent sporting exercise or frequent sporting exercise plus other recreational activities). An additional question asked respondents to report their engagement in muscular strength/endurance training. This included lifting weights, doing push-ups and using exercise machines. Participants were classified as participating or not participating in muscular strength and endurance training.

Participants also completed a six-item version of the Duke Activity Status Index (DASI), which was used as a measure of general fitness. The DASI was developed to measure functional capacity and strongly correlates with peak oxygen uptake (r = 0.80) (Hlatky et al., 1989). The index comprised of questions regarding ability to participate in six activities of increasing intensity, weighted according to the MET value for each activity. A total score was calculated by adding the weighted scores for the six items.

## 4 Outcome measures

### 4.1 Anthropometric measures

Height (cm), weight (kg), waist circumference (WC) (cm), mid-upper arm circumference and triceps skinfold thickness (mm) were measured as previously described (Wannamethee et al., 2007). WC was used as a measure of obesity using a sex-specific cut point (> 102 cm) (Anon, 1998). Muscle mass was derived from the mid-upper arm muscle circumference (MAMC) using the formula mid-upper arm circumference − (0.3142 × triceps skinfold thickness) (Miller et al., 2002).

### 4.2 Physical function and strength

Gait speed (m/s) was derived from a 3-metre walking test and was used as a measure of physical function. Grip strength (kg) was used as a marker of muscular strength and was measured using a Jamar Hydraulic Hand Dynamometer. Participants had three attempts for each hand and the highest score was used.

### 4.3 Definitions of sarcopenia and sarcopenic obesity

Sarcopenia and severe sarcopenia were defined using the European Working Group on Sarcopenia in Older People (EWGSOP) definition (Cruz-Jentoft et al., 2010). Both conditions required (i) low muscle mass (participants in the lowest two-fifths of the MAMC distribution were classified as having low muscle mass) and either (ii) low grip strength (< 30 kg) or (iii) low gait speed (≤ 0.8 m/s) (Cruz-Jentoft et al., 2010). Severe sarcopenia required the presence of all three conditions (Cruz-Jentoft et al., 2010). To determine sarcopenia-obesity groups, men with sarcopenia and severe sarcopenia were collapsed into an overall sarcopenic group. They were then categorised into four groups: non-sarcopenic non-obese (WC ≤ 102 cm, not sarcopenic), sarcopenic non-obese (WC ≤ 102 cm, sarcopenic), non-sarcopenic obese (WC > 102 cm, not sarcopenic), or sarcopenic obese (WC > 102 cm, sarcopenic).

## 5 Covariates

Men self-reported the following: social class, derived from longest held occupation; doctor diagnosis of medical conditions including angina, heart attack, heart failure, other heart conditions, bronchitis, depression, emphysema, osteoporosis, Parkinson's disease, cancer (excluding skin cancers), arthritis and stroke; cigarette smoking habits and alcohol intake. Number of medical conditions was categorised as low (< 3) or high (≥ 3) according to the above medical conditions. Cigarette smoking was classified as current or recent smokers (given up in the last ten years), ex-smokers (gave up > 10 years ago) and never smokers. Alcohol intake was classified as high (> 15) or low (≤ 15 units of alcohol per week).

## 6 Statistical analysis

Descriptive statistics of demographic, physical health and PA variables selected a priori were calculated firstly for sarcopenia groups and then for sacropenic obese groups. Differences between groups were examined using one-way ANOVA and chi-square tests. Mean and standard deviation (SD) of PA levels were calculated for quintiles of MAMC, WC, grip strength and gait speed. Quintiles were generated based on participants with available data on the variable of interest after excluding participants without valid accelerometry data and those living in care homes (sample size, n = 1521). Tests for linear trend were conducted by entering each component of sarcopenia/sarcopenic obesity (muscle mass, MAMC [cm]; obesity, WC [cm]; physical performance, gait speed [m/s]; muscular strength, grip strength [kg]) continuously into regression models, adjusting for age and WC. Multinomial logistic regression was used to examine the association between PA and sarcopenia categories. Exposure measures were objectively measured PA variables (sedentary time [min/day], number of breaks in sedentary time per hour, LPA [min/day], MVPA [min/day]) and strength training (y/n). The primary outcomes were (i) sarcopenia, (ii) severe sarcopenia and (iii) sarcopenic obesity. The “non-sarcopenic” and “non-sarcopenic non-obese” groups were selected as reference categories in respective analyses. In an additional analysis including just obese men, we compared the non-sarcopenic obese group with the sarcopenic obese group by selecting the former as the reference category. Analyses were initially adjusted for age (years), wear time, season and region (model 1); and then for occupational social class, number of medical conditions, smoking, alcohol and height (model 2). Model 2 also controlled for WC in analyses investigating risk of sarcopenia and severe sarcopenia. Final models (model 3) also controlled for the other intensities of PA i.e. MVPA was included as a covariate when examining associations of sedentary time, breaks in sedentary time and LPA. All analyses were carried out using STATA version 12 (Stata Corp, College Station, TX).

### 6.1 Sensitivity analyses

We repeated models excluding men with heart attack, heart failure or stroke (*n* = 206). MVPA was positively skewed so we repeated models using square-root-transformed MVPA to normalise the distribution. We also repeated analyses using self-reported PA and DASI fitness scores as exposures.

## 7 Results

**Fig. 1:**
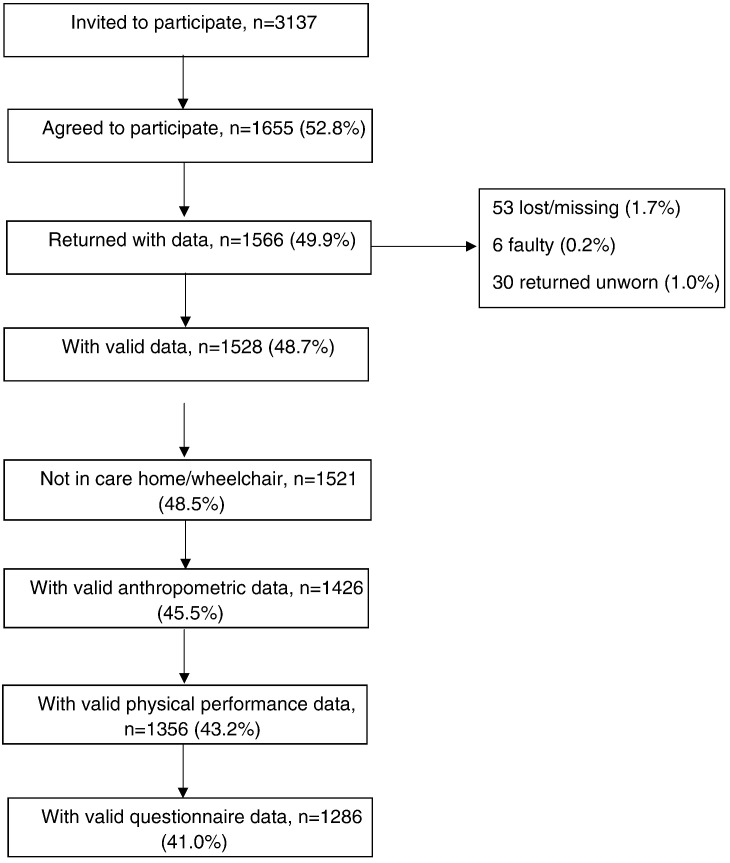
Recruitment flow chart – BRHS, 2010–12.

1655/3137 (52.8%) of survivors agreed to take part in the physical examination and wear an accelerometer. After excluding men who were confined to a wheelchair or resident in a care home (n = 7), 1521 (48.5%) men provided valid PA data, of whom 1286 (41.0%) had complete data from the physical examination, functional tests and questionnaire (Fig. 1). Participants not attending this wave of data collection or with incomplete data had a higher BMI ten years earlier (27.0 vs. 26.7 kg/m^2^, p < 0.05), were less active (40.9% vs. 60.3% at least moderately active, p < 0.001), were more likely to smoke (20.2% vs. 11.5%, p < 0.001) and were older (69.8 years vs. 66.0 years, p < 0.001) than those included in the study sample (n = 1286).

183 men (14.2%) were classified as sarcopenic, 70 (5.4%) as severely sarcopenic and 1033 (80.3%) as non-sarcopenic (Table 1). Sarcopenic and severely sarcopenic men were older, shorter, had more chronic conditions, lower body weight, BMI and WC, and were generally less physically active than the reference group. The severely sarcopenic group were the least active with 19.8 min/day of MVPA compared to 42.1 min/day in the non-sarcopenic group.

Table 2 presents participant characteristics according to sarcopenia-obesity groups. 64 men (5.0%) were classified as sarcopenic-obese, 189 (14.7%) as sarcopenic non-obese, 442 (34.4%) as non-sarcopenic obese and 591 (46%) as non-sarcopenic non-obese. 25.3% of individuals with sarcopenia/severe sarcopenia were also obese. Sarcopenic obese and sarcopenic non-obese were older, shorter, had more chronic conditions and were less active than the non-sarcopenic non-obese group. The sarcopenic obese group was the least active.

Table 3 presents participant PA levels by quintiles of MAMC, WC, grip strength and gait speed. None of the PA variables were associated with MAMC after adjustments. WC was inversely associated with MVPA, LPA, breaks in sedentary time and participation in muscle strengthening/endurance exercises and positively associated with sedentary time after adjusting for age. Gait speed and grip strength were positively associated with MVPA and inversely associated with sedentary time. Gait speed was also positively associated with LPA and breaks in sedentary time. Participation in muscle strengthening/endurance exercises was associated with higher grip strength but this did not remain significant after adjusting for WC and age.

In multinomial logistic regression models, none of the PA variables were associated with risk of sarcopenia but they were associated with severe sarcopenia (Table 4). Each extra 30 min of sedentary time was associated with an increased risk of severe sarcopenia, which persisted after adjusting for social class, number of chronic conditions, smoking, alcohol, height and WC, but was fully attenuated after adjusting for MVPA. Conversely, each extra 30 min of MVPA and LPA were associated with reduced risk of severe sarcopenia. Associations remained significant after adjusting for social, health and lifestyle factors, but LPA was no longer associated after adjusting for MVPA. The association between MVPA and a reduced risk of severe sarcopenia remained significant after adjusting for sedentary time. Associations between strength training and severe sarcopenia were not interpretable due to low numbers with severe sarcopenia who did strength training.

Table 5 reports the risk of sarcopenic obesity categories by PA levels. Sedentary time was associated with an increased risk of sarcopenic obesity independent of MVPA. Sedentary breaks, LPA and MVPA were associated with reduced risk of sarcopenic obesity in final adjusted models. Similar associations were observed between PA variables and the risk of non-sarcopenic obesity. Strength training was associated with a reduced risk of non-sarcopenic obesity and non-obese sarcopenia, although the association with non-obese sarcopenia was of borderline significance (p = 0.06). Associations between strength training and sarcopenic obesity were also uninterpretable due to low numbers participating in strength training. In an additional analysis including just obese men, the non-sarcopenic obese category was selected as the reference category and compared with the sarcopenic obese group (Supplementary Table 1). After adjusting for social, health and lifestyle factors, sedentary time was associated with an increased risk of sarcopenic obesity (RR 1.16, 95% confidence interval [CI] 1.02, 1.33) and MVPA was associated with a reduced risk (RR 0.60, 95% CI 0.39, 0.95). Associations were not significant after adjusting for other PA intensities.

### 7.1 Sensitivity analyses

Excluding men with heart attack, heart failure or stroke and using square-root-transformed MVPA did not alter our conclusions (data not shown). Self-reported PA and DASI fitness scores were also associated with severe sarcopenia, non-sarcopenic obesity and sarcopenic obesity. They were also associated with the sarcopenic and sarcopenic non-obese groups in final adjusted models (Supplementary Tables 2-3).

## 8 Discussion

In this sample of free-living older British men, MVPA was associated with a reduced risk of severe sarcopenia, independent of sedentary time and WC. Similarly, MVPA and to a lesser extent LPA and sedentary breaks were independently associated with a reduced risk of sarcopenic obesity. Conversely, sedentary time was associated with an increased risk of sarcopenic obesity independent of MVPA. Analyses between PA and the individual components of sarcopenia and sarcopenic obesity showed that PA was not associated with muscle mass alone, suggesting that PA may be more important for preserving a healthy weight, physical performance and muscular strength in older men.

Prevalence of sarcopenia and sarcopenic obesity was lower than that of previous studies with this cohort when they were younger (Atkins et al., 2014a). However, the prevalence of sarcopenia and sarcopenic obesity was comparable with previous waves data when we used the same definition (data not shown) (Atkins et al., 2014a), suggesting that the discrepancies mainly reflect our use of the EWGSOP diagnostic criteria for sarcopenia (Cruz-Jentoft et al., 2010), including objective measures of gait speed and grip strength that were previously unavailable. Nevertheless, sarcopenia was still common in the present sample and comparable with the prevalence in other cohorts of a similar age (Kim et al., 2013, Gianoudis et al., 2015).

To our knowledge, this is the first study to explore associations of PA with sarcopenia, severe sarcopenia and sarcopenic obesity using objectively measured rather than self-reported PA. Higher MVPA was associated with significant reductions in the risk of severe sarcopenia and sarcopenic obesity. Our conclusions appear consistent with previous studies using self-report and objective measures of PA (Ryu et al., 2013, Atkins et al., 2014b, Shephard et al., 2013). In our study, each additional 30 min per day of MVPA was associated with a 47% reduction in severe sarcopenia risk. This is comparable with a recent longitudinal study also using objectively measured PA; older Japanese men taking > 9000 steps per day had approximately half the risk of sarcopenia compared to men taking < 6700, with additional reduction in risk for PA > 3 METS (Shephard et al., 2013). However, direct comparisons between these studies are difficult due to the different definitions used for sarcopenia and different devices used to measure PA. A number of studies defining sarcopenia with muscle mass alone have demonstrated an association between PA and sarcopenia (Atkins et al., 2014b, Ryu et al., 2013, Shephard et al., 2013). In this population, only when muscle mass was combined with low muscular strength and low physical performance (severe sarcopenia) were associations observed. Furthermore, none of the PA variables were associated with muscle mass alone, but there were associations with gait speed, grip strength and WC. Our findings are consistent with a recent study that also showed no association between PA and muscle mass in men (Kim et al., 2015) and a number of other studies showing positive associations between PA and muscular strength in older populations (Hamer and Stamatakis, 2013, Kuh et al., 2005). Potentially PA may be more important for preserving muscular strength and physical function than muscle mass in elderly men. Moreover, there is a body of evidence suggesting that muscular strength is more important than muscle mass for cardiometabolic health and physical function (Stephen and Janssen, 2009, Visser et al., 2000). An alternative explanation may be that participants who had sarcopenia at previous waves are more likely to have dropped out of the study due to the increased risk of mortality, as previously reported in this cohort (Atkins et al., 2014a). Thus our sample may be unrepresentative of the lower end of muscle mass distribution and therefore reducing our power to detect associations between physical activity and muscle mass.

For the first time we demonstrated an association between objectively measured PA and a reduced risk of sarcopenic obesity. This is consistent with studies using self-reported PA (Ryu et al., 2013) and fitness measures (Pedrero-Chamizo et al., 2015). Notably, we showed that even LPA and sedentary breaks were associated with a reduced risk of sarcopenic obesity, which appears to be driven by higher gait speed and lower WC. Higher sedentary time was associated with increased risk of sarcopenic obesity, independent of MVPA, which is broadly consistent with other studies that have shown independent associations between sedentary behaviour with obesity (Healy et al., 2008, Jakes et al., 2003) and sarcopenia (Gianoudis et al., 2015) as individual outcomes. As expected, PA was strongly associated with a reduced risk of obesity (Atkins et al., 2014a). PA was also associated with a reduced risk of sarcopenic obesity when compared to those with just obesity, suggesting that the presence of both conditions heightens associations with PA. Importantly, the ‘non-sarcopenic non-obese’ reference group demonstrate healthier behaviours than the ‘non-sarcopenic’ reference group, which may explain why associations between PA and sarcopenic obesity are more pronounced than those with sarcopenia and severe sarcopenia.

Self-reported strength training was associated with a reduced risk of non-obese sarcopenia and non-sarcopenic obesity. Participation in strength training was most strongly associated with WC and grip strength. Previous studies in similar populations have also shown positive associations between PA and grip strength (Kuh et al., 2005, Hamer and Stamatakis, 2013). A recent systematic review showed that resistance training interventions are particularly effective for improving muscular strength in aging adults (Cruz-Jentoft et al., 2014). Participation in strength training was low in this sample, which may have reduced our power to detect these associations.

One of the main strengths of this study is the use of objective measures of PA. This allowed us to investigate whether sedentary time, breaks in sedentary time and LPA were associated with these conditions. Our findings contribute evidence to the discussion about whether PA guidelines for older people should also encourage sedentary breaks and LPA. Whilst MVPA may be most strongly related to outcomes, even increasing LPA and sedentary breaks may be beneficial and also more feasible in this population. Another strength is the use of muscle mass, muscular strength and physical performance to define sarcopenia. According to the EWGSOP criteria these are essential components in the diagnosis of sarcopenia (Cruz-Jentoft et al., 2010). Although there are more precise measures of muscle mass such as dual X-ray absorptiometry, MAMC is a practical and easy clinical measure of muscle mass that is readily available in primary care settings and is recognised as a valid measure by the American Heart Association (Cornier et al., 2011). Our sample is from a large cohort of community-dwelling men rather than an at risk population, increasing the generalisability of the results. However, our findings may not be generalizable to women and non-white ethnic groups. Sarcopenia and sarcopenic obesity have also been associated with increased risk of all-cause mortality in this cohort (Atkins et al., 2014a). Consequently participants who had sarcopenia at previous waves are more likely to have dropped out, potentially leading to underestimation of the true associations with activity levels. Further, our sample generally displayed healthier behaviours than those excluded from the analysis and therefore may have been more active than the general population, potentially attenuating observed associations. The cut points we used for defining MVPA are well established and have been validated in older adults (Copeland & Esliger, 2009). Actigraph accelerometers are less accurate at differentiating between sedentary behaviour and LPA. Therefore, some sedentary time may include standing time. Nevertheless, Actigraph-measured sedentary time correlates strongly with ActivPal measured sitting time (r = 0.76) (Healy et al., 2011), which is a reliable measure for sitting. Finally, this investigation is a cross-sectional study, and causality cannot be definitely determined.

## 9 Conclusion

Objectively measured PA was associated with a reduced risk of severe sarcopenia and sarcopenic obesity. PA at a moderate-to-vigorous intensity may be the most favourable for reducing risk; however, LPA and breaking up prolonged periods of sedentary behaviour may also reduce the risk of sarcopenic obesity. Further longitudinal studies are required to determine the causality of these associations.

## Author contributions

DA analysed the data and drafted the initial manuscript. BJJ conceived the idea and design of the manuscript, raised funding for the study and participated in its design and data acquisition. CS and OP contributed to making the database. SGW and PHW raised funding for the study and participated in its conception, design, data acquisition, coordination and interpretation. SA and LL collected and downloaded the data and helped interpret the results. All authors made contributions to the interpretation and drafting of the final article.

## Transparency Document

Transparency document.

## Figures and Tables

**Table 1:** Participant characteristics of subjects with sarcopenia, severe sarcopenia and without sarcopenia defined according to MAMC, gait speed and grip strength – BRHS, 2010–12.

Characteristic	N	Non-sarcopenia	Sarcopenia	Severe sarcopenia	Between group differences (p value)^a^
N, (%, n)	1286	80.3% (1033)	14.2% (183)	5.4% (70)	

*Sociodemographics*					
Age, mean ± SD	1286	77.6 ± 4.1	79.7 ± 4.7	83.1 ± 5.2	p < 0.001
Current/recent smoker, (%, n)	1286	7.6 (78)	9.8 (18)	7.1 (5)	0.556
Moderate or heavy drinkers, (%, n)	1286	14.0 (145)	9.8 (18)	15.7 (11)	0.266
Manual workers, (%, n)	1286	44.8 (463)	44.8 (82)	57.1 (40)	0.255

*Physical health*					
CHD^b^ (%, n)	1286	15.5 (160)	19.1 (35)	15.7 (11)	0.465
Height (cm), mean ± SD	1286	172.1 ± 6.4	170.1 ± 7.0	167.0 ± 5.7	p < 0.001
Weight (kg), mean ± SD	1286	81.4 ± 11.9	72.8 ± 11.6	72.5 ± 12.3	p < 0.001
BMI (kg/m^2^), mean ± SD	1286	27.5 ± 3.7	25.1 ± 3.5	26.0 ± 4.2	p < 0.001
Waist circumference (cm), mean ± SD	1286	100.9 ± 10.7	94.5 ± 10.0	97.3 ± 12.1	p < 0.001
MAMC (cm), mean ± SD	1286	25.5 ± 2.7	21.8 ± 1.9	21.6 ± 2.1	p < 0.001
Hand grip (kg), mean ± SD	1286	32.3 ± 9.9	28.7 ± 10.1	22.2 ± 6.1	p < 0.001
Gait speed (m/s), mean ± SD	1286	0.95 ± 0.2	0.82 ± 0.2	0.62 ± 0.1	p < 0.001
Chronic conditions ≥ 3, (%, n)	1286	11.5 (119)	17.5 (32)	18.6 (13)	*p* < 0.05

*PA levels (mean, 95% CI)*					
Time sedentary (min/day)	1286	610.9 (606.0, 615.7)	614.1 (602.1, 626.1)	650.6 (632.0, 669.2)	p < 0.001
Breaks in sedentary time per hour	1286	7.3 (7.2, 7.4)	7.3 (7.0, 7.6)	6.6 (6.0, 7.1)	p < 0.05
Time in LPA (min/day)	1286	201.9 (198.1, 205.6)	196.4 (187.1, 205.7)	169.2 (152.5, 185.9)	p < 0.001
Time in MVPA (min/day)	1286	42.1 (40.1, 44.0)	37.9 (32.8, 43.1)	19.8 (14.4, 25.1)	p < 0.001
Wear time (min/day)	1286	854.8 (850.8, 858.8)	848.4 (838.3, 858.5)	839.5 (821.1, 857.9)	0.105
Participation in muscle strengthening/endurance exercises, (%, n)	1090^c^	12.9% (115)	10.1% (15)	7.6% (4)	0.352

CHD, coronary heart disease; BMI, body mass index; MAMC, mid-upper arm muscle circumference; MVPA, moderate-to-vigorous physical activity; LPA, light physical activity; CPM, counts per minute.

a x^2^ for percentages, analysis of variance for means.

b Men with CHD consisted of men reporting a doctor diagnosis of a heart attack heart failure or stroke.

c Smaller sample size due to lower response on self-reported data.

**Table 2:** Participant characteristics according to Sarcopenic Obesity Groups – BRHS, 2010–12.

Characteristic	N	Non-sarcopenic non-obese	Sarcopenic non-obese	Non-sarcopenic obese	Sarcopenic obese	Between group differences (p value)^a^
N, (%, n)	1286	46.0% (591)	14.7% (189)	34.4% (442)	5.0% (64)	

*Sociodemographics*						
Age, mean ± SD	1286	77.7 ± 4.0	80.8 ± 5.2	77.5 ± 4.2	80.0 ± 4.6	p < 0.001
Current/recent smoker, (%, n)	1286	6.8 (40)	9.0 (17)	8.6 (38)	9.4 (6)	0.605
Moderate or heavy drinkers, (%, n)	1286	12.9 (76)	10.1 (19)	15.6 (69)	15.6 (10)	0.255
Manual workers, (%, n)	1286	40.8 (241)	41.3 (78)	50.2 (222)	68.8 (44)	p < 0.001

*Physical health*						
CHD^b^ (%, n)	1286	15.4 (91)	20.1 (38)	15.6 (69)	12.5 (8)	0.368
Height (m), mean ± SD	1286	171.5 ± 6.2	169.0 ± 7.0	173.0 ± 6.5	170.0 ± 6.3	p < 0.001
Weight (kg), mean ± SD	1286	74.4 ± 7.3	68.0 ± 8.5	90.9 ± 10.2	86.6 ± 8.7	p < 0.001
BMI (kg/m^2^), mean ± SD	1286	25.3 ± 2.2	23.8 ± 2.5	30.4 ± 3.2	30.0 ± 2.8	p < 0.001
Waist circumference (cm), mean ± SD	1286	93.6 ± 6.1	90.6 ± 7.1	110.6 ± 7.0	109.3 ± 6.4	p < 0.001
MAMC (cm), mean ± SD	1286	24.8 ± 2.7	21.5 ± 2.1	26.4 ± 2.5	22.4 ± 1.5	p < 0.001
Hand grip (kg), mean ± SD	1286	32.5 ± 9.7	26.8 ± 10.2	32.0 ± 10.1	27.2 ± 7.9	p < 0.001
Gait speed (m/s), mean ± SD	1286	0.99 ± 0.2	0.79 ± 0.2	0.91 ± 0.2	0.72 ± 0.2	p < 0.001
Chronic conditions ≥ 3, (%, n)	1286	8.6 (51)	17.5 (33)	15.4 (68)	18.8 (12)	p < 0.05

*PA levels (mean, 95% CI)*						
Time sedentary (mins per day)	1286	599.1 (592.8, 605.5)	616.0 (604.1, 627.9)	626.6 (619.3, 633.9)	648.3 (629.1, 667.6)	p < 0.001
Breaks in sedentary time per hour	1286	7.7 (7.5, 7.9)	7.4 (7.1, 7.7)	6.8 (6.7, 7.0)	6.2 (5.7, 6.7)	p < 0.001
Time in LPA (mins per day)	1286	211.4 (206.3, 216.4)	197.5 (188.2, 206.7)	189.2 (183.7, 194.7)	163.4 (146.8, 180.1)	p < 0.001
Time in MVPA (mins per day)	1286	48.5 (45.8, 51.1)	36.9 (31.8, 42.1)	33.6 (30.9, 36.2)	21.0 (15.9, 26.1)	*p* < 0.001
Wear time (mins per day)	1286	858.9 (853.8, 864.0)	850.4 (840.7, 860.1)	849.3 (843.0, 855.6)	832.8 (812.5, 853.0)	p < 0.05
Participation in muscle strengthening/endurance exercises (%, n)	1090^c^	15.6 (80)	7.0 (11)	9.3 (35)	18.2 (8)	*p* < 0.05

CHD, coronary heart disease; BMI, body mass index; MAMC, mid-upper arm muscle circumference; MVPA, moderate-to-vigorous physical activity; LPA, light physical activity; CPM, counts per minute.

a x^2^ for percentages, analysis of variance for means.

b Men with CHD consisted of men reporting a doctor diagnosis of a heart attack heart failure or stroke.

c Smaller sample size due to lower response on self-reported data.

**Table 3:** Physical activity levels across quintiles of components of sarcopenia and sarcopenic obesity (n = 1286) – BRHS, 2010–12.

		Quintiles of sarcopenia and sarcopenic obesity components							
MAMC (cm)	≤ 22.11 (n = 258)	22.11–24.21 (n = 264)	24.21–25.74 (n = 254)	25.74–27.34 (n = 253)	≥ 27.34 (n = 257)				

	Mean (SD)	Mean (SD)	Mean (SD)	Mean (SD)	Mean (SD)	Β (95% CI)^a^	p value for trend (unadjusted)	Β (95% CI)^a^	p value for trend (WC and age adjusted)
Time sedentary (min/day)	611.3 (84.5)	613.4 (78.5)	611.3 (80.0)	616.7 (78.9)	614.9 (79.5)	0.02 (− 0.04, 0.08)	0.580	− 0.01 (− 0.06, 0.05)	0.821
Breaks in sedentary time/hour	7.5 (2.1)	7.4 (2.1)	7.3 (2.0)	7.1 (1.9)	7.2 (2.0)	− 0.12 (− 0.20, − 0.03)	< 0.05	− 0.02 (− 0.10, 0.05)	0.558
Time in LPA (min/day)	199.5 (64.0)	200.9 (65.9)	200.1 (59.9)	194.6 (61.0)	201.2 (62.9)	− 0.03 (− 0.11, 0.05)	0.474	0.04 (− 0.04, 0.11)	0.351
Time in MVPA (min/day)^b^	32.4 (42.3)	32.1 (41.4)	32.1 (40.1)	32.1 (35.1)	35.3 (41.1)	− 0.01 (− 0.07, 0.06)	0.843	0.02 (− 0.04, 0.09)	0.530
Participation in muscle strengthening/endurance exercises (%)	12.3	12.2	12.9	10.2	13.8	0.15 (− 0.38, 0.69)	0.574	0.17 (− 0.32, 0.65)	0.503

Waist circumference (cm)	≤ 91.15 (n = 264)	91.15–96.65 (n = 251)	96.65–101.80 (n = 257)	101.80–108.65 (n = 254)	≥ 108.65 (n = 260)				

	Mean (SD)	Mean (SD)	Mean (SD)	Mean (SD)	Mean (SD)	Β (95% CI)^a^	p value for trend (unadjusted)	Β (95% CI)^a^	p value for trend (age adjusted)
Time sedentary (min/day)	612.8 (86.4)	591.4 (76.1)	604.5 (75.1)	621.5 (80.7)	636.5 (75.2)	0.64 (0.42, 0.86)	< 0.001	0.74 (0.52, 0.96)	< 0.001
Breaks in sedentary time/hour	7.6 (2.2)	7.8 (2.0)	7.4 (2.0)	7.1 (1.9)	6.4 (1.8)	− 1.37 (− 1.66, − 1.09)	< 0.001	− 1.51 (− 1.80, − 1.22)	< 0.001
Time in LPA (min/day)	204.9 (66.6)	213.9 (58.7)	205.4 (61.8)	199.0 (63.1)	173.8 (55.8)	− 1.13 (− 1.41, − 0.85)	< 0.001	− 1.29 (− 1.57, − 1.01)	< 0.001
Time in MVPA (min/day)^b^	38.2 (40.7)	44.6 (52.0)	33.7 (38.1)	27.0 (37.0)	22.1 (32.8)	− 1.03 (− 1.26, − 0.80)	< 0.001	− 1.36 (− 1.60, − 1.11)	< 0.001
Participation in muscle strengthening/endurance exercises (%)	14.8	11.8	14.1	11.1	9.5	− 1.72 (− 3.69, 0.24)	0.086	− 2.08 (− 4.04, − 0.11)	< 0.05

Gait speed (m/s)	≤ 0.73 (n = 249)	0.73–0.86 (n = 270)	0.86–0.97 (n = 259)	0.97–1.10 (n = 257)	≥ 1.10 (n = 251)				

	Mean (SD)	Mean (SD)	Mean (SD)	Mean (SD)	Mean (SD)	Β (95% CI)^a^	p value for trend (unadjusted)	Β (95% CI)^a^	p value for trend (WC and age adjusted)
Time sedentary (min/day)	636.1 (80.2)	624.2 (81.4)	613.4 (83.8)	603.2 (71.0)	590.2 (76.4)	− 0.02 (− 0.02, − 0.01)	< 0.001	− 0.01 (− 0.02, − 0.01)	< 0.001
Breaks in sedentary time/hour	6.6 (2.2)	7.0 (2.0)	7.4 (1.8)	7.7 (1.9)	7.8 (2.0)	0.03 (0.02, 0.03)	< 0.001	0.02 (0.01, 0.02)	< 0.001
Time in LPA (min/day)	172.2 (68.9)	189.5 (63.0)	203.5 (55.9)	211.1 (57.7)	220.2 (56.4)	0.03 (0.03, 0.04)	< 0.001	0.02 (0.02, 0.03)	< 0.001
Time in MVPA (min/day)^b^	13.2 (24.3)	26.6 (34.9)	34.1 (38.4)	37.6 (40.9)	47.7 (36.4)	0.04 (0.03, 0.04)	< 0.001	0.03 (0.02, 0.03)	< 0.001
Participation in muscle strengthening/endurance exercises (%)	9.3	12.8	12.9	14.6	11.5	0.03 (− 0.02, 0.07)	0.209	0.00 (− 0.04, 0.04)	0.846

Grip strength (kg)	≤ 22 (n = 253)	22–30 (n = 282)	30–34 (n = 246)	34–40 (n = 317)	≥ 40 (n = 248)				

	Mean (SD)	Mean (SD)	Mean (SD)	Mean (SD)	Mean (SD)	Β (95% CI)^a^	p value for trend (unadjusted)	Β (95% CI)^a^	p value for trend (WC and age adjusted)
Time sedentary (min/day)	622.6 (84.2)	622.8 (80.1)	607.8 (78.8)	610.8 (81.5)	599.2 (71.7)	− 0.35 (− 0.56, − 0.14)	< 0.05	-0.20 (− 0.41, 0.01)	0.062
Breaks in sedentary time/hour	7.1 (2.1)	7.1 (2.0)	7.4 (2.1)	7.4 (1.9)	7.5 (1.9)	0.31 (0.03, 0.58)	< 0.05	0.14 (− 0.14, 0.42)	0.329
Time in LPA (min/day)	193.8 (63.8)	189.4 (63.8)	202.2 (62.3)	202.6 (60.7)	212.1 (61.5)	0.41 (0.15, 0.67)	< 0.05	0.21 (− 0.06, 0.48)	0.125
Time in MVPA (min/day)^b^	23.9 (36.1)	24.9 (37.9)	35.4 (41.6)	37.5 (38.6)	46.2 (39.1)	0.79 (0.57, 1.00)	< 0.001	0.58 (0.34, 0.82)	< 0.001
Participation in muscle strengthening/endurance exercises (%)	10.3	11.2	10.5	14.2	15.8	1.86 (0.05, 3.68)	< 0.05	1.19 (− 0.59, 2.97)	0.189

MVPA, moderate-to-vigorous physical activity; CPM, counts per minute; LPA, light physical activity.

a Linear trend regression coefficient. For regression analyses time sedentary, in light and in MVPA was converted to units of 30 min/day.

b Values expressed as median and interquartile range; MVPA was transformed by square rooting for regression analyses.

**Table 4:** Multinomial logistic regression analysis of sarcopenia and severe sarcopenia risk according to physical activity levels (reference: non-sarcopenic group) – BRHS, 2010–12.

	Non-sarcopenic	Sarcopenic	Severe sarcopenic
	RR (95% CI)	RR (95% CI)	RR (95% CI)
Model 1^a^			
Sedentary time (30 min/day)	1.00	0.99 (0.92, 1.05)	1.19 (1.06, 1.35)
Breaks in sedentary time (breaks/hour)	1.00	1.05 (0.96, 1.14)	0.92 (0.80, 1.05)
Light PA time (30 min/day)	1.00	1.02 (0.93, 1.11)	0.85 (0.73, 0.99)
MVPA time (30 min/day)	1.00	1.03 (0.87, 1.21)	0.49 (0.32, 0.76)
Strength training (no/yes)^e^	1.00	0.89 (0.50, 1.59)	

Model 2^b^			
Sedentary time (30 min/day)	1.00	1.03 (0.96, 1.11)	1.22 (1.07, 1.38)
Breaks in sedentary time (breaks/hour)	1.00	0.97 (0.89, 1.06)	0.88 (0.76, 1.02)
Light PA time (30 min/day)	1.00	0.98 (0.89, 1.07)	0.84 (0.72, 0.98)
MVPA time (30 min/day)	1.00	0.92 (0.77, 1.10)	0.45 (0.29, 0.72)
Strength training (no/yes)^e^	1.00	0.82 (0.45, 1.50)	

Model 3			
Sedentary time (30 min/day)^c^	1.00	1.01 (0.92, 1.12)	1.07 (0.91, 1.26)
Breaks in sedentary time (breaks/hour)^c^	1.00	0.99 (0.90, 1.08)	0.96 (0.83, 1.12)
Light PA time (30 min/day)^c^	1.00	0.99 (0.90, 1.09)	0.93 (0.79, 1.10)
MVPA time (30 min/day)^d^	1.00	0.93 (0.73, 1.19)	0.53 (0.30, 0.93)

Models 1 and 2 included only one activity variable as an exposure per regression whereas model 3 also controlled for other intensities of physical activity.

MVPA, moderate-to-vigorous physical activity; LPA, light physical activity; CPM, counts per minute; RR, risk ratio.

a Model 1 adjusted for age, wear time, season and region.

b Model 2 additionally adjusted for social class, number of chronic conditions, smoking, alcohol, height and waist circumference.

c Model 3 further adjusted for MVPA.

d Model 3 further adjusted for sedentary time.

e Risk ratios were not interpretable due to low participation in strength training in the severe sarcopenic group.

**Table 5:** Multinomial logistic regression analysis of sarcopenic obesity risk according to physical activity levels (reference: non-sarcopenic non-obese group) – BRHS, 2010–12.

	Non-sarcopenic non-obese	Sarcopenic non-obese	Non-sarcopenic obese	Sarcopenic obese
	RR (95% CI)	RR (95% CI)	RR (95% CI)	RR (95% CI)
Model 1^a^				
Sedentary time (30 min/day)	1.00	1.04 (0.96, 1.12)	1.23 (1.16, 1.30	1.43 (1.26, 1.62)
Breaks in sedentary time (breaks/hour)	1.00	1.00 (0.91, 1.09)	0.79 (0.74, 0.85)	0.71 (0.61, 0.83)
Light PA time (30 min/day)	1.00	0.97 (0.89, 1.07)	0.83 (0.77, 0.89)	0.71 (0.60, 0.82)
MVPA time (30 min/day)	1.00	0.91 (0.76, 1.09)	0.58 (0.50, 0.67)	0.33 (0.21, 0.51)
Strength training (no/yes)^e^	1.00	0.50 (0.26, 0.99)	0.56 (0.36, 0.85)	

Model 2^b^				
Sedentary time (30 min/day)	1.00	1.02 (0.95, 1.10)	1.22 (1.16, 1.30)	1.40 (1.23, 1.59)
Breaks in sedentary time (breaks/hour)	1.00	1.00 (0.92, 1.09)	0.80 (0.74, 0.86)	0.73 (0.63, 0.85)
Light PA time (30 min/day)	1.00	0.99 (0.90, 1.09)	0.83 (0.77, 0.89)	0.73 (0.62, 0.85)
MVPA time (30 min/day)	1.00	0.95 (0.79, 1.13)	0.58 (0.50, 0.68)	0.34 (0.21, 0.53)
Strength training (no/yes)^e^	1.00	0.52 (0.26, 1.03)	0.54 (0.35, 0.83)	

Model 3				
Sedentary time (30 min/day)^c^	1.00	1.00 (0.90, 1.11)	1.11 (1.02, 1.20)	1.18 (0.99, 1.40)
Breaks in sedentary time (breaks/hour)^c^	1.00	1.02 (0.92, 1.12)	0.86 (0.80, 0.93)	0.84 (0.71, 0.99)
Light PA time (30 min/day)^c^	1.00	1.00 (0.90, 1.11)	0.91 (0.84, 0.98)	0.85 (0.72, 1.01)
MVPA time (30 min/day)^d^	1.00	0.94 (0.74, 1.21)	0.69 (0.57, 0.84)	0.47 (0.27, 0.84)

Models 1 and 2 included only one activity variable as an exposure per regression whereas model 3 also controlled for other intensities of physical activity.

MVPA, moderate-to-vigorous physical activity; LPA, light physical activity; CPM, counts per minute; RR, risk ratio.

a Model 1 adjusted for age, wear time, season and region.

b Model 2 additionally adjusted for social class, number of chronic conditions, smoking, alcohol and height.

c Model 3 further adjusted for MVPA.

d Model 3 further adjusted for sedentary time.

e Risk ratios were not interpretable due to low participation in strength training in the sarcopenic obese group.

## Appendix A Supplementary data

Supplementary tables. Image 1
