# Supplementary material for: Cross-sectional associations of objectively measured physical activity and sedentary time with sarcopenia and sarcopenic obesity in older men
Source: Prev Med. 2016 Oct;91:264–72. doi: 10.1016/j.ypmed.2016.08.040 (PMC5061552; doi:10.1016/j.ypmed.2016.08.040)
Supplement: Supplementary file 2 — Supplementary tables. [file mmc2.doc]

**Supplementary table 1.** Multinomial logistic regression analysis of sarcopenic obesity risk according to physical activity levels (reference: non-sarcopenic obese group) – BRHS, 2010-12

| **Model 1^a^** | | |
| --- | --- | --- |
|  | **Non-sarcopenic obese** | **Sarcopenic obese** |
|  | RR (95% CI) | RR (95% CI) |
| Sedentary time (30 min/day) | 1.00 | 1.18 (1.03, 1.35) |
| Breaks in sedentary time (breaks/hour) | 1.00 | 0.90 (0.77, 1.05) |
| Light PA time (30 min/day) | 1.00 | 0.85 (0.72, 0.99) |
| MVPA time (30 min/day) | 1.00 | 0.60 (0.38, 0.93) |
| **Model 2^b^** | | |
| Sedentary time (30 min/day) | 1.00 | 1.16 (1.02, 1.33) |
| Breaks in sedentary time (breaks/hour) | 1.00 | 0.91 (0.78, 1.06) |
| Light PA time (30 min/day) | 1.00 | 0.86 (0.73, 1.02) |
| MVPA time (30 min/day) | 1.00 | 0.60 (0.39, 0.95) |
| **Model 3** | | |
| Sedentary time (30 min/day)^c^ | 1.00 | 1.08 (0.90, 1.29) |
| Breaks in sedentary time (breaks/hour)^c^ | 1.00 | 0.96 (0.82, 1.14) |
| Light PA time (30 min/day)^c^ | 1.00 | 0.93 (0.77, 1.11) |
| MVPA time (30 min/day)^d^ | 1.00 | 0.71 (0.40, 1.27) |

Models 1 and 2 included only one activity variable as an exposure per regression whereas model 3 also controlled for other intensities of physical activity.

^a^ Model 1 adjusted for age, wear time, season and region.

^b^ Model 2 additionally adjusted for social class, number of chronic conditions, smoking, alcohol and height

^c^ Model 3 further adjusted for MVPA

^d^ Model 3 further adjusted for sedentary time

^e^ Risk ratios were not interpretable due to low participation in strength training in the sarcopenic obese group

MVPA, moderate-to-vigorous physical activity; LPA, light physical activity; CPM, counts per minute; RR, risk ratio

**Supplementary table 2.** Multinomial logistic regression analysis of sarcopenia and severe sarcopenia risk according to self-reported physical activity and fitness levels (reference: non-sarcopenic group) – BRHS, 2010-12

|  | **Model 1^a^** | | | |
| --- | --- | --- | --- | --- |
|  |  | **Non-sarcopenic** | **Sarcopenic** | **Severe Sarcopenic** |
|  | n | RR (95% CI) | RR (95% CI) | RR (95% CI) |
| DASI fitness score^c^ | 1130 | 1.00 | 0.98 (0.97, 1.00) | 0.93 (0.91, 0.95) |
| Self-reported PA^d^ | 1225 | 1.00 | 0.89 (0.79, 1.00) | 0.77 (0.63, 0.94) |
|  | **Model 2^b^** | | | |
| DASI fitness score^c^ | 1130 | 1.00 | 0.97 (0.96, 0.99) | 0.92 (0.89, 0.94) |
| Self-reported PA^d^ | 1225 | 1.00 | 0.83 (0.74, 0.94) | 0.75 (0.61, 0.93) |

^a^ Model 1 adjusted for age, wear time, season and region

^b^ Model 2 additionally adjusted for social class, number of chronic conditions, smoking, alcohol, height and waist circumference

^c^ DASI fitness score was a continuous variable. A higher score represents higher fitness levels.

**^d^** Self-reported PA was treated as a continuous variable with six categories (inactive, occasional, light, moderate, moderately vigorous and vigorous)

**Supplementary table 3.** Multinomial logistic regression analysis of sarcopenic obesity risk according to self-reported physical activity and fitness levels (reference: non-sarcopenic non-obese group) – BRHS, 2010-12

| **Model 1^a^** | | | | | |
| --- | --- | --- | --- | --- | --- |
|  |  | **Non-sarcopenic non-obese** | **Sarcopenic non-obese** | **Non-sarcopenic obese** | **Sarcopenic obese** |
|  | n | RR (95% CI) | RR (95% CI) | RR (95% CI) | RR (95% CI) |
| DASI fitness score^c^ | 1130 | 1.00 | 0.97 (0.95, 0.98) | 0.96 (0.95, 0.97) | 0.92 (0.90, 0.94) |
| Self-reported PA^d^ | 1225 | 1.00 | 0.83 (0.73, 0.94) | 0.76 (0.70, 0.83) | 0.62 (0.50, 0.76) |
| **Model 2^b^** | | | | | |
| DASI fitness score^c^ | 1130 | 1.00 | 0.97 (0.96, 0.98) | 0.97 (0.95, 0.98) | 0.92 (0.90, 0.94) |
| Self-reported PA^d^ | 1225 | 1.00 | 0.84 (0.74, 0.95) | 0.77 (0.70, 0.84) | 0.64 (0.51, 0.79) |

^a^ Model 1 adjusted for age, wear time, season and region

^b^ Model 2 additionally adjusted for social class, number of chronic conditions, smoking, alcohol and height

^c^ DASI fitness score was a continuous variable. A higher score represents higher fitness levels.

**^d^** Self-reported PA was treated as a continuous variable with six categories (inactive, occasional, light, moderate, moderately vigorous and vigorous)
